# Endocrine Disrupting Chemicals: An Occult Mediator of Metabolic Disease

**DOI:** 10.3389/fendo.2019.00112

**Published:** 2019-03-01

**Authors:** Olga Papalou, Eleni A. Kandaraki, George Papadakis, Evanthia Diamanti-Kandarakis

**Affiliations:** ^1^Department of Endocrinology & Diabetes, Hygeia Hospital, Athens, Greece; ^2^STEPS Stoffwechselzentrum, Biel/Bienne, Switzerland

**Keywords:** obesity, insulin resistance, human metabolism, endocrine disrupting chemical (EDC), environmental contaminants, obesogens, enviromental chemicals, diabetes mellitus

## Abstract

Endocrine disrupting chemicals (EDCs), a heterogeneous group of exogenous chemicals that can interfere with any aspect of endogenous hormones, represent an emerging global threat for human metabolism. There is now considerable evidence that the observed upsurge of metabolic disease cannot be fully attributed to increased caloric intake, physical inactivity, sleep deficit, and ageing. Among environmental factors implicated in the global deterioration of metabolic health, EDCs have drawn the biggest attention of scientific community, and not unjustifiably. EDCs unleash a coordinated attack toward multiple components of human metabolism, including crucial, metabolically-active organs such as hypothalamus, adipose tissue, pancreatic beta cells, skeletal muscle, and liver. Specifically, EDCs' impact during critical developmental windows can promote the disruption of individual or multiple systems involved in metabolism, via inducing epigenetic changes that can permanently alter the epigenome in the germline, enabling changes to be transmitted to the subsequent generations. The clear effect of this multifaceted attack is the manifestation of metabolic disease, clinically expressed as obesity, metabolic syndrome, diabetes mellitus, and non-alcoholic fatty liver disease. Although limitations of EDCs research do exist, there is no doubt that EDCs constitute a crucial parameter of the global deterioration of metabolic health we currently encounter.

## Introduction

During the last 50 years, the global rates of obesity, diabetes, and metabolic disease have increased exponentially (1). Based on the data from the *International Diabetes Federation*, ~415 million people worldwide were estimated to have diabetes in 2015, a percentage that will rise to 642 million people by 2040 (2), while, simultaneously, in 2016, the *World Health Organization* estimated that 650 million people are obese and ~2 billion people are overweight worldwide (3). When these numbers are translated to individual morbidity and mortality, the calculated societal and financial burden we are facing is hugely disappointing (4–6). Thus, in order to tackle this burgeoning metabolic disease epidemic, we have to identify the underlying pathogenetic factors and mitigate their deleterious impact.

Genetic background, increased caloric intake, physical inactivity, sleep deficit, and aging have been recognized by medical community as major pathogenetic parameters in metabolic disease (7). However, existing bibliography suggests that the observed upsurge of metabolic disease cannot be fully attributed to the above-mentioned risk factors. In fact, individuals nowadays tend to weigh more than they did 20–30 years ago even when the amount of activity and caloric intake are the same (8).

Among environmental factors involved in the worldwide deterioration of metabolic health, endocrine disrupting chemicals (EDCs) have drawn the biggest attention of scientific community, and not unjustifiably (9). In fact, the documented increase of obesity and metabolic disease correlates and coincides chronically with an upsurge in EDCs generation and widespread use (10, 11). While emerging epidemiological data are highlighting the close association between EDCs and metabolic disease epidemic, experimental data, and animal models have postulated multiple potent pathways by which EDCs alter hormonal milieu and promote metabolic disease, mandating immediate action and policy-making.

In this review, we will present evidence of how environmental contaminants can perturb human metabolism, through interfering with control of energy metabolism and targeting multiple metabolically crucial organs, causing ultimately an altered balance toward obesity and dysmetabolism and contributing to this global metabolic emergency.

## Endocrine-Disrupting Chemicals (EDCs): Have we Opened the Pandora's Box?

### Overview of Endocrine-Disrupting Chemicals–Historical Data

Endocrine-disrupting chemicals (EDCs) are defined as exogenous chemicals or mixtures of chemicals that interfere with any aspect of endogenous hormonal signaling, affecting not only production, release, and transport of hormones, but also their cellular metabolism, binding action, and elimination (9, 12). It is a heterogeneous, rapidly growing group of natural or man-made chemical compounds, including synthetic chemicals used as industrial solvents, plastics, plasticizers, fungicides, pesticides, heavy metals, and pharmaceutical agents (12, 13).

While scientific community was slowly gaining knowledge regarding environmental contaminants, it was in 1991 that the term “endocrine-disrupter” was firstly introduced (14).

In 2006, researchers at the University of California, Irvine, highlighted the role of EDCs in the global obesity epidemic and coined the term “obesogen” (15). Obesogens were defined as environmental agents that have the ability to promote obesity via inducing an increment in the number of fat cells and/or the storage of fat in adipocytes, as well as via shifting metabolism toward a mode of caloric storage (16). As the list of obesogenic chemicals is continuously growing, the obesogen field has broadened in recognizing chemicals that are linked with diabetes and other metabolic diseases (17). Thus, in 2015, the Parma consensus statement proposed the term “metabolism-disrupting chemicals (MDCs)” to describe the EDCs that can promote diabetes, obesity, and fatty liver, through perturbing metabolism at multiple cellular levels (18).

**Figure 1:**
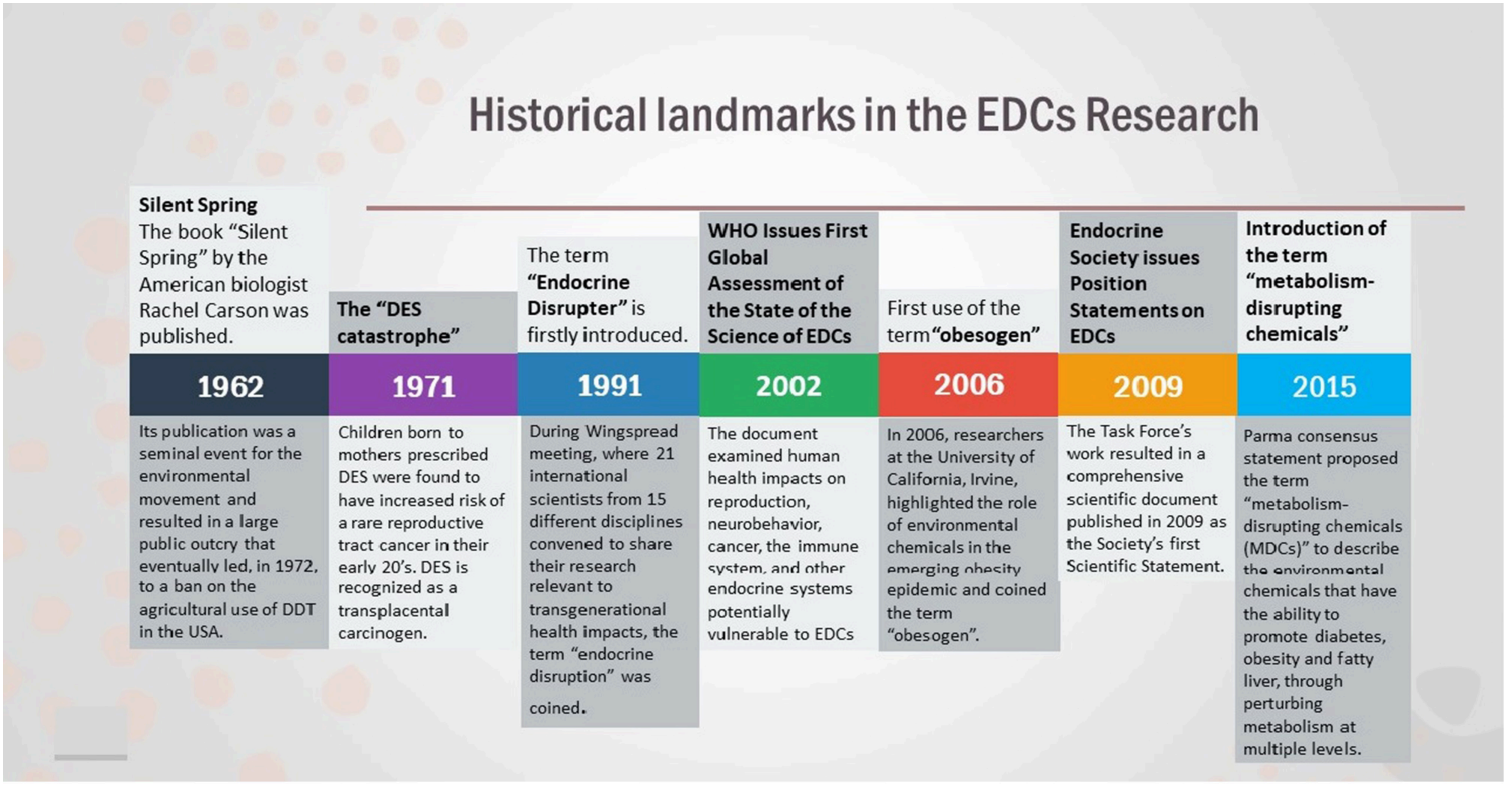
Historical landmarks in the field of EDCs Research.

Overall, EDCs have ascended as a global health priority and organizations such as Endocrine Society and the WHO/UNEP have issued official statements regarding the putative health risks posed by EDCs, describing the plethora of diseases EDCs are related to, including reproduction, neurodevelopment, thyroid, metabolism, and hormone-related cancers (9, 19). Although we have made a considerable progress in understanding EDCs, we are still looking at the “tip of the iceberg” and there are much more to be learned (Figure 1).

### EDCs Characteristics and Unique Properties

Over 1,000 synthesized chemical compounds are considered to be EDCs, such as plastics (bisphenol A), plasticizers (phthalates), industrial solvents/lubricants, and their byproducts (polychlorinated biphenyls, polybrominated biphenyls, dioxins), pesticides (methoxychlor, chlorpyrifos, dichlorodiphenyltrichloroethane), fungicides (vinclozolin), and pharmaceutical agents (diethylstilbestrol) (20). There are multiple routes of exposure to the above EDCs, including air, water, food, and consumer products. Some of them have low accumulation in human body (BPA, phthalates), while others are very lipophilic, accumulating easily in the food chain and the adipose tissue (persistent organic pollutants - POPs) (Table 1) (21). EDCs can also be found in various biological fluids, including sera, urine, amniotic fluid, and breast milk (22).

**Table 1:** Endocrine disrupters (EDCs) with documented metabolism-disrupting effects.

**Endocrine disrupters (EDCs)**	**Description and characteristics**
**PERSISTENT ORGANIC POLLUTANTS (POPS)**	
Dichlorodiphenyltrichloroethane (DDT)	A synthetic insecticide with a long half-life, extensive use, and lipophilic nature. The United States banned DDT in 1972 due to its effects on the environment and human health. DDT and its metabolites seem to contribute to the manifestation of endocrine-related diseases, including diabetes mellitus.
Dioxins	Dioxins are mainly by-products of industrial processes but can also result from natural processes, such as volcanic eruptions and forest fires. Their half-life in the body is estimated to be 7 to 11 years. They accumulate in food chain and in the adipose tissue of human body. The most harmful dioxin is 2,3,7,8-tetrachlorodibenzo -p-dioxin (TCDD).
Polychlorinated biphenyls (PCBs)	Man-made synthetic chemical mixtures, widely used in electrical equipment, ink solvents and especially plasticizers until the late 1970s, after which time they were banned. Their use has been associated with the obesity epidemic.
Perfluorinated compounds (PFCs)	PFCs have been detected in food packaging, furniture, clothes, cookware, and non-stick surfaces in order to repel grease and oil. They have been linked with obesity and adipose tissue dysfunction.
Polybrominated flame retardants	They have been used in a variety of materials, such as furniture, electronics, and construction materials, as flame retardants. Via accumulating in the environment and human fat tissue, these man-made chemicals have been linked with adverse health outcomes, including obesity.
**NON-PERSISTENT EDCS**	
Bishenol A (BPA)	A synthetic organic compound, mainly used as plasticizer, is commonly detected in water bottles, food containers, and metal-based cans. The magnitude of human exposure to this EDC is depicted to the observation that ~93% of Americans have measurable urine levels of BPA. It is characterized by a rapid metabolization to its non-bioactive forms and a short half-life (4–5 h in adult humans).
Phthalates	Pthalates have been widely used in the manufacture of polyvinyl chloride plastics and vinyl products. As a result, they have been detected in multiple household products, including pacifiers, children's toys, food packaging, medical devices, and furnishings. Animal models have displayed a close interrelationship between phthalates and metabolic disease.
Tributyltin	An organotin commonly used as a heat stabilizer and as fungicide. It can also be found in house dust. Although data on human exposures are scarce, it has been detected in human liver and blood.

Although EDCs are characterized as a group of compounds with high heterogeneity, there are some key characteristics that define them and enable us to better understand their mechanism of action and their consequences.

- EDCs, just like hormones, may promote disrupting effects even in very low levels of exposure, particularly if exposure takes place in a critical developmental period. In fact, EDCs display a non-monotonic (U-shaped or biphasic) response, which means that low doses may have a much stronger impact in human body than higher doses (23, 24).
- EDCs usually display a much lower affinity for hormone receptors, compared to natural ligands. For instance, the affinity of the estrogen receptors (ER), ER-a and ER-b, for one of the most widespread EDCs, bisphenol-A (BPA) is 1,000–10,000 fold lower than for 17b-estradiol (E2) (25). Nevertheless, EDCs, even under these circumstances, can have detrimental effects in several human tissues.
- Time of exposure is critical in EDCs' effects. EDC exposure during sensitive developmental periods, such as fetal life, infancy, puberty, pregnancy, and menopause, can detrimentally affect individuals and predispose them to a multitude of diseases (26, 27).
- There is a lag between the time of exposure to EDCs and the clinical expression of a disease, suggesting that the repercussions of EDCs exposure may not be directly evident, but may be ultimately manifested many years after the exposure (12).
- Since environmental pollution is not caused by a single compound, it is rather reasonable that humans are constantly exposed not to one, but to a cocktail of EDCs. In a mixture, the different classes of EDCs interact in an either additive or synergistic way, making even more difficult not only to predict the net effect they provoke, but also to evince a cause-and-effect association between a specific EDC and an associated effect-disease (28).

### Vulnerable Windows of Susceptibility–Developmental Programming and Transgenerational Effects of EDCs

The “Developmental origins of health and diseases” (DOHaD) hypothesis, initially expressed by David Barker, has introduced the concept that early life growth and development is vulnerable to environmental disruptors, which can determine the risk for health and disease (29). In other words, environmental disruption during critical developmental windows is capable of promoting subtle changes in gene expression and biological molecular processes, which, ultimately, alter permanently the developmental trajectory and lead to long-lasting dysfunction.

Nutrition has been introduced as a powerful environmental stimulus that can promote intrauterine modifications, manifesting later in life as increased vulnerability to obesity and dysmetabolism. More analytically, undernutrition *in utero* and low-birth weight, combined with early catch-up growth during infancy, was shown to be correlated with augmented risk for impaired metabolism, cardiovascular disease and reproductive deregulation in adulthood (30–32). Analogously, maternal obesity or obesogenic maternal diet during gestation, was associated with increased oxidative stress in the offspring, making them sensitive to diabetogenic effects (33, 34).

Apart from nutrition, EDCs also hold a special position in the DOHaD hypothesis. The “Diethylstilbestrol (DES) catastrophe” provided the original proof regarding the ability of EDCs to perturb developmental processes and predispose to certain diseases. Back in 1940–1970, prescription of DES to numerous women, in order to prevent miscarriage, led to reproductive tract anomalies and substantially increased the incidence of mammary cancer in their offspring (35). Nowadays, accumulating data support that EDCs impact during critical developmental windows can be disruptive for multiple systems involved in human metabolism. For example, both in animal and human studies, developmental exposure to DES, led to increased weight gain and adipocyte hyperplasia in the offspring, predisposing them to obesity during adulthood (36, 37). Likewise, EDCs acting during fetal or perinatal period, can permanently perturb adipose tissue function, via altering the programming of mesenchymal stem cells (38).

One of the main mechanisms, via which EDCs alter programming of cell and tissue differentiation, is by inducing epigenetic changes (9, 39). Epigenetic changes are defined as heritable alterations in gene expression, without any structural change in DNA sequence, which can be transmitted through mitosis and/or meiosis. There are several mechanisms, by which epigenetics can modulate gene expression and modify gene transcription, including methylation of cytosine residues on DNA, post-translational modification of histones, and altered microRNA expression (40, 41).

**Figure 2:**
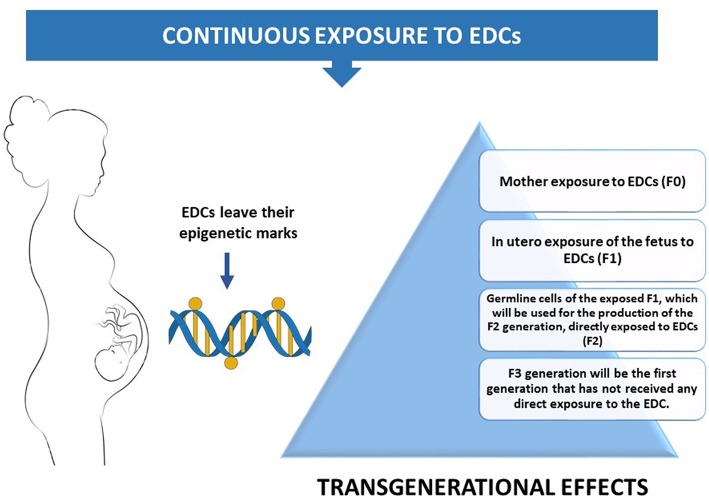
A pregnant mother's exposure to the environment can inadvertently introduce EDCs into the exquisitely calibrated hormonal milieu of the embryo.

Adult exposure to EDCs is likely a potential factor of adverse health outcomes. However, when this exposure takes place in early life development, EDCs-induced epigenetic alterations permanently affect the epigenome in the germline, enabling changes to be transmitted to the next generations (42). This transgenerational component of EDCs' can be applied only when exposure occurs during development. As soon as a pregnant female (F0) is exposed to an EDC, germline cells of her fetus (F1) are also exposed to it. These germline cells of the exposed F1 will be the gametes of the F2 generation, resulting in the direct exposure of the F2 generation to this EDC. F3 generation will be the first generation that has not been directly exposed to the EDC (43). Therefore, if the effects of the EDC persist in F3 generation, they considered to be transgenerational. (Figure 2).

Transgenerational sequelae of EDCs have been best studied in BPA and vinclozolin. Specifically, in animal models, prenatal exposure to BPA led to alterations in the prostate epigenome, affecting genes that are interrelated with prostate cancer (44, 45), while ancestral environmental exposure to vinclozolin in rodents was shown to be associated with transgenerational effects on the development of physiological, neural, and behavioral phenotypes in adulthood in the F3 generation(46, 47).

Regarding metabolism, experimental data about potential transgenerational effects of EDCs are now emerging. For example, prenatal TBT exposure via drinking water of pregnant F0 animals led to increased most white adipose tissue (WAT) depot weights, adipocyte size, and adipocyte number, and reprogrammed MSCs toward the adipocyte lineage at the expense of bone in all three subsequent generations(48). Skinner et al. have shown that a mixture of plastic derived compounds, BPA and phthalates, can promote epigenetic transgenerational alterations that predispose offspring in the F3 generation to obesity (49).

Overall, although the precise molecular mechanisms of how EDCs promote epigenetic changes remain unclear and cause and effect data are lacking, it is very likely that these chemicals have a more deleterious impact in human endocrine system than robust data can support so far. Even if we manage to annihilate environmental contaminants today, the impact of their disruptive effects to the next generations would be probably an after event documentation.

## Human Metabolism: a Precise Hormonal Interplay Between Tissues

Energy homeostasis involves the coordinated homeostatic regulation of food intake (energy inflow) and energy expenditure (energy outflow). A precise orchestration of the actions of metabolically-active, such as liver, pancreas, adipose tissue, brain, gut, and thyroid, stands in the core of human energy homeostasis

Hypothalamus has a critical role in regulating energy intake and appetite, decoding neural influences arising from other sites of the brain as well as hormonal signals. There are two different neural populations that coexist in the arcuate nucleus (ARC) of hypothalamus and exert antagonistic effects: neuropeptide Y (NPY) and agouti-related protein (AgRP)-expressing neurons with orexigenic actions, whereas anorexigenic effects are expressed by proopiomelanocortin (POMC), cocaine expressing neurons and amphetamine-regulated transcript protein (CART)-expressing neurons (50).

More importantly, the gastrointestinal (GI) tract and particularly GI regulatory peptides, constitutes another benchmark for the regulation of energy homeostasis (51). Nutrient ingestion triggers gut peptides secretion, such as cholecystokinin (CCK), glucagon-like peptide-1 (GLP-1), and peptide YY (PYY), which via either activation of local neuronal circuits or endocrine signaling directly in the CNS, establishes a gut–brain axis (52, 53). Simultaneously, accumulating evidence suggests that any alteration in the microbiota composition can lead to an imbalanced production of metabolites and substances involved in the performance of physiological functions, and ultimately promote metabolic diseases, such as obesity and diabetes (54).

Adipose tissue constitutes one of the key regulators of energy homeostasis. As an endocrine gland with central role in nutrient metabolism, adipose tissue dysfunction stands in the pathophysiological “heart” of metabolic disease (55, 56). Adipocytes, the primary cells of adipose tissue, derive from mesenchymal stem cells (MSCs). Transformation of an MSC into an adipocyte requires initial commitment to the adipose lineage, followed by terminal differentiation into a mature adipocyte, where PPAR-γ pathway constitutes the master regulator of adipogenesis (57). Apart from mature adipocytes, the balance and the stage of receptor profile of other cell types in adipose tissue, including stem cells, preadipocytes, macrophages, neutrophils, lymphocytes, and endothelial cells, has a pivotal role in maintaining the control of energy homeostasis (58–60).

Via its multiple hormones, mainly glucagon and insulin, pancreas is the key regulator of glucose homeostasis. Increased exogenous glucose levels, after a meal, stimulate insulin secretion in β-cells. Specifically, glucose taken up by beta cells undergoes intermediary metabolism, promoting an increase in the ATP/ADP ratio and the closure of plasma membrane ATP-sensitive K+(KATP) channels. This induced cellular depolarization promotes insulin release from the cells, which enters the circulation and interacts with receptors in target-organs, initiating the insulin-mediated glucose disposal (61). Impairment of any of the above stages can lead to inadequate glucose-mediated insulin release and ultimately lead to diabetes. Apart from its central role in glucose regulation, strong evidence suggests that insulin, together with leptin, can influence hypothalamic control of energy homeostasis. However, the extent and nature of this co-interaction has to be further clarified (62). Converse to the actions of insulin, glucagon is secreted in response to low levels of blood glucose, in order to increase glucose production by stimulating glycogenolysis and gluconeogenesis by the liver. Glucagon seems to be also implicated in food intake and satiety. Preliminary data has shown that glucagon administration ameliorates the sense of hunger and diminishes food intake in humans and rats, confirming that glucagon specifically decreases meal size owing to increased satiation (63).

Skeletal muscle can be described as the traffic controller of the metabolic circulation. Via regulating about 80% of post-prandial insulin-stimulated glucose disposal skeletal muscle constitutes the starting point of energy production (64). As a pure energy-producing organ, it is full of mitochondria that also have a regulative role in energy homeostasis. Glucose transport in skeletal muscle is mediated through insulin, which triggers the recruitment of the glucose transporter GLUT4 to the plasma membrane. Insulin also activates the necessary enzymes (hexokinase and glycogen synthase) to enhance glycogen synthesis. When calorie intake exceeds energy expenditure, ample concentrations of energy substrates accumulate intracellularly in skeletal muscle. Increased glucose entry results in augmented glycolytic flux and glucose oxidation, leading ultimately to increased oxidative stress and metabolic deregulation (65).

Finally, liver constitutes the main glucose storage of human body, playing an important role both in anabolism and catabolism. It also stands in the heart of the metabolic interconnection of key organs, including skeletal muscle and adipose tissue (63). During fasting, liver is responsible for generating glucose as a fuel for the human body. After an increase in the pancreatic hormone glucagon, the cascade of kinase action that releases glucose from the stored glycogen via glycogenolysis is initiated in liver. As soon as glycogen is depleted, *de novo* glucose synthesis from lactate or glycerol undertakes the major role for the generation of glucose as a fuel for other tissues (66–68).

## Human Metabolism Under Attack—Effect of EDCs in Metabolically-Active Organs

### Hypothalamus

EDCs may have a modulatory role in disrupting normal feeding behavior through interfering with the hypothalamic—hindbrain circuits and by directly interacting with steroid and nuclear receptors. Particularly, when EDC exposure begins early in life, *in utero* or perinatally, when the formation of these brain circuits takes place, the EDCs metabolic impact may be much greater for the feeding behavior (52).

BPA, a well-known EDC with estrogenic properties, has been highlighted as a potent disrupter of hypothalamic feeding circuitry, via multiple mechanisms. Firstly, acting *in utero*, neonatal exposure of female rats to BPA led to downregulation of hypothalamic ARC ER-a protein levels, known to exert anorexigenic effects (53). In another experimental model, perinatal exposure to BPA was found to adversely modulate the development of POMC system in the ARC in a sexually differentiated way. Specifically, early-life exposure to the obesogen BPA altered the expression of the genes encoding ER, NPY, POMC, and AgRP and decreased POMC fiber density in the paraventricular nucleus during adulthood, when offspring followed a high-fat diet, demonstrating that BPA can make them more vulnerable to manifesting diet-induced obesity and metabolic dysfunction (69). The same research group has also shown that perinatal exposure of mice to BPA or diethylstilbestrol (DES) at environmentally relevant doses can also perturb leptin actions to hypothalamus. In fact, mice exposed to these EDCs were more resistant to leptin-induced suppression of food intake, body weight loss, and hypothalamic pro-opiomelanocortin (POMC) upregulation, permanently altering the neurobiology of metabolic homeostasis (70).

Furthermore, data in bibliography suggest that *in utero* exposure to BPA can affect offspring not only during adulthood but can also lead to transgenerational effects, through inducing epigenetic changes in gene expression and DNA methylation of imprinted genes in the brain (71). Finally, BPA can also alter energy intake through inducing compulsive eating behavior. In this context, Sullivan et al. have shown that perinatal exposure of primates to BPA led to a significant change in the number of tyrosine hydroxylase-immunoreactive neurons in brain regions, supporting the hypothesis that BPA alters affective behaviors and hedonic feeding (58).

Among other EDCs, TCDD exposure during adulthood in a rodent model resulted in reduced food and water intake and altered macronutrient preference, via changes in the hypothalamic-pituitary-adrenal axis, the melanocortin neurocircuitry, and the neuropeptides that control fluid intake (59). Chronic exposure of pregnant mice to TBBPA inhibited the transcriptional activity of TSH-releasing hormone and melanocortin receptor 4 (MC4R) in the hypothalamus, which is a major regulator of energy homeostasis, with its mutations causing obesity (60). Finally, adult exposure to TBT in mice promoted profound alteration of the leptin-NPY-NPY-Y1 receptor system (72).

Recently, disruption of circadian rhythm, not only in hypothalamus but also in other tissues such as liver, has been identified as novel metabolic risk factor, with EDCs emerging as contributors to disease risk in this area (73). For example, tolyfluanid was shown to negatively affect normal circadian feeding patterns in mice (74), while studies in male zebrafish demonstrated that BPA can alter circadian activity (75).

Concluding, although data are still indicatory and sometimes controversial, we do have sufficient evidence regarding the involvement of EDCs in the regulation of food behavior in the brain that warrants further investigation by the scientific community.

### Adipose Tissue

In view of the emerging epidemiological data linking EDCs exposure with obesity, experimental studies have focused on adipose tissue. In fact, adipose tissue can accumulate lipophillic EDCs and become the principal target tissue of obesogens. A variety of compounds has been shown to modulate adipocyte physiology, including insulin action and adipokine secretion, alter adipocyte differentiation and induce chronic inflammation. This EDCs-induced adipocyte dysfunction may be a contributing factor to the epidemic of metabolic disease (76).

Firstly, EDCs can promote adipogenesis, via disrupting fat cell differentiation and development. One of the first studies demonstrating it included an animal model of male C57BL/6 mice exposed to TBT, which displayed enhanced gene expression of the adipogenic markers CCAAT enhancer binding protein-β (C/EBPβ) and sterol regulatory element-binding protein-1 (Srebp1). In utero exposure to TBT resulted in augmented adipose mass in 10-week old males (66). Similarly, in another experimental model, PCBs—exposed adult male C57BL/6 mice exhibited increased body weight gain. This effect was found to be dependent on the aryl hydrocarbon receptor (AhR), as AhR-null mice did not show the same PCB-induced increase in body weight (66). Apart from the above, BPA, lead, PCB-126, atrazine, the fungicide triflumizole and organophosphate insecticides have also been incriminated as potent promoters of weight gain and adiposity in various animal models with variable levels of exposure (67).

PPARγ pathway disruption has been highlighted as one of the well-studied mechanisms by which EDCs promote adipogenesis. MSC up-regulation and preadipocyte differentiation into adipocytes have been shown to be triggered by numerous EDCs, such as DDT, BPA, phthalates, and PCBs [reviewed in (67)]. However, all the above EDCs differ structurally, indicating that their effects in adipocyte differentiation are potentiated through distinct pathways. A respectable share of EDCs elicits their adipogenic effects, through targeting PPARγ. EDCs can lead to enhanced adipogenesis either via directly binding and activating downstream cascades, or via increasing PPARγ expression, they allow for a lower threshold [reviewed in (68)]. Perinatal exposure to 4-nonylphenol (4-NP) resulted in an increase in PPARγ gene expression and sterol regulatory element-binding factor 1 (SREBF-1) expression in adipose tissue, affecting ultimately adipogenesis (77).

Another nuclear hormone receptor with a catalytic role in adipogenesis is the glucocorticoid receptor (GR). In an experimental model by Sargis RM, various EDCs were evaluated regarding their ability to stimulate the GR and drive adipogenesis in the 3T3-L1 cell lines. Among them, BPA, phthalates, and tolylfluanid (TF) were found to promote 3T3-L1 differentiation, through GR activation (78). Specifically, TF has been highlighted as a “structurally unique environmental glucocorticoid” actively implicated in GR signaling, as this fungicide has the ability to displace radiolabeled glucocorticoid from the GR (79). Finally, apart from the direct stimulation of the receptor, glucocorticoid signaling is also controlled by the interconversion of glucocorticoids between active and inactive states through the enzymatic action of 11β-hydroxysteroid dehydrogenase type 1 and 2 (11β-HSD-1/2). EDCs can act at this level, as well. For example, BPA also promotes GR-mediated indirect effects by increasing mRNA expression and enzymatic activity of 11β-HSD1 (80).

Apart from the effects in the adipocyte differentiation, EDCs can induce alterations in adipocyte endocrine function, via interfering with the mechanisms of action of key metabolic hormones. For example, TF exposure can attenuate insulin signal transduction via especially down-regulating insulin receptor substrate-1 (IRS-1) levels (81), while, simultaneously, it can imitate the binding of corticosterone to the GR and enhance insulin-induced lipogenesis (79). Furthermore, multiple experimental and animal models have shown that EDCs can modulate the synthesis and release of adipokines. For example, BPA can increase levels of leptin (82), decrease adiponectin secretion *in vitro* (83) and levels of adiponectin in mice offspring after *in utero* exposure (84), and enhance the release of IL-6 and TNF from human adipocytes (85). This disruption in the release of multiple signaling molecules can negatively affect local and systemic energy homeostasis, leading to inflammation, insulin resistance, dyslipidemia, and ultimately to metabolic dysfunction.

### Pancreas

Environmental contaminants can negatively affect multiple aspects of β-cell physiology, including beta cell function and survival, insulin release, and glucose disposal. Dioxin exposure, mainly TCDD, was one of the first to be linked with metabolic alterations in multiple experimental studies. Specifically, TCDD was demonstrated to decrease glucose uptake in pancreas and impair insulin secretion (86). Through promoting continuous insulin release, TCDD exposure led to the consumption of cellular insulin reservoir and ultimately β-cell “exhaustion” (87), suggesting that insulin deficiency may ensue after sustained exposure to this compound. The adverse metabolic impact of TCDD has been also documented in human observational studies. In a longitudinal study of veterans exposed to TCDD during the Vietnam War, serum TCDD exposure was clearly interrelated with the prevalence of T2DM and insulin resistance in this population (88).

Among other EDCs, oral administration of TBT was shown to inhibit the proliferation and induce the apoptosis of islet cells via multiple pathways, causing a decrease of relative islet area in the animals treated for 60 days, which could result in a disruption of glucose homeostasis (89). Arsenic, another environmental pollutant that contaminates drinking water, can also impair insulin secretion, via downregulating insulin gene expression (90) and interfering with calpain-10-mediated proteolysis and activation of SNAP-25, a key step in insulin granule exocytosis (91). Indeed, epidemiological studies have confirmed the link between arsenic exposure and diabetes, with arsenic being correlated specifically with indices of β-cell dysfunction or decreased insulin secretion, more powerfully than with indices of insulin resistance (92).

BPA has also been investigated as a potent disrupter of beta cell function. Starting from *in utero*, an experimental model showed that pregnant mice treated with the BPA during gestation, at environmentally relevant doses, exhibit profound glucose intolerance and altered insulin sensitivity as well as increased body weight several months after delivery, mainly through impairments in beta-cell function and mass (93). Furthermore, *in vivo* experiments suggest that BPA exposure augments insulin release and glucose stimulated insulin secretion, in an estrogen receptor-a (ERa) dependent manner (94). Sex steroids, except for their primary reproductive role, exert key effects on metabolic target tissues, including pancreas, controlling β-cell insulin secretion in both cGMP-dependent and independent pathways. Thus, BPA can exert part of its metabolic effects in pancreas via estrogen-dependent pathways (95). In accordance with all the above, urinary BPA concentration in US adults were shown to be correlated with augmented β-cell function hyperinsulinemia and insulin resistance, particularly in men (96).

Recently, oxidative and endoplasmatic reticulum (ER) stress have been highlighted as crucial pathogenetic mechanisms of diabetes (97). In an experiment by Maechier P et al., β-cells exposed to hydrogen peroxide activated the production of p21 cyclin-dependent kinase inhibitor and decreased insulin mRNA, ATP and calcium flux reductions in mitochondria and cytosol (98). Furthermore, as shown by Tiedge et al. β-cells are lower in antioxidant enzymes levels (superoxide dismutase, catalase and glutathione peroxidase) and more sensitive to ROS adverse actions (99). Oxidative stress can significantly compromise β cell function, as pancreatic β cells are innately more sensitive. Several EDCs including BPA, arsenic and DEHP can disrupt β-cell function via promoting oxidative stress (68). For instance, rats exposed to phenolic compounds octylphenol, nonylphenol, and BPA displayed disrupted islet morphology and β-cell function, mainly via alterations in mitochondrial architecture and gene expression (100). Analogously, long-term exposure to BPA triggered spontaneous insulinitis in non-obese diabetic (NOD) mice, a model of immune-mediated diabetes, suggesting that BPA can accelerate the exhaustion of β-cell reserve via immune modulations in pancreatic islets. As it becomes obvious, the immunomodulatory effects of BPA in this animal model suggest that EDCs might also possibly contribute to the increasing T1DM prevalence (101).

### Skeletal Muscle

In addition to data demonstrating that EDCs disrupt insulin production and beta cell function, an increasing body of evidence suggests that peripheral insulin action is also compromised. In fact, human exposure to various EDCs has been causally correlated with insulin resistance, such as BPA, TCDD, and phthalates (68). EDCs can disrupt insulin action via altering the expression or impairing the activity of multiple insulin signaling intermediates, including the insulin receptor, insulin receptors substrates, phosphatidylinositol-3-kinase (PI3k) - Protein kinase B (Akt) pathway and glucose transporters (102). For instance, BPA exposed rodents displayed glucose intolerance and global insulin resistance, due to disrupted insulin signaling, via defects in phosphorylation of both the insulin receptor and Akt (102, 103).

### Liver

Except for skeletal muscle, liver is equally critical in orchestrating peripheral insulin actions and, therefore, for predicting metabolic risk. Among the environmental parameters that can have an adverse impact in the liver, EDCs have been widely highlighted, as they can catalytically perturb hepatic function. More analytically, EDCs have the ability to affect liver physiology and metabolism either indirectly, via the peripheral effects of adipose tissue dysfunction and pancreatic insulin release, or directly via autonomous effects in liver cells. The net effect of both of these actions is promoting lipogenesis, liver steatosis and ultimately non-alcoholic fatty liver disease (NAFLD) (104).

In pancreatic β cells, EDCs might increase or decrease insulin production, affecting indirectly hepatic lipogenesis, via down- or upregulating, through SREBP1C, the gene expression of various lipogenic enzymes. (105). Simultaneously, once an EDC enters the liver, it can bind to specific nuclear hormone receptors in liver cells (106). After EDC binding to these receptors, co-regulator proteins (either co-activators or co-repressors) are recruited and modulate gene expression of proteins involved in lipid homeostasis and/or the reprogramming of the epigenome. In the literature, various animal models have investigated the effect of EDC exposure in liver physiology, leading to the conclusion that EDCs can directly promote increased hepatic lipid accumulation and NAFLD (107–109).

### Novel Players in the Metabolism Disruption by EDCs (Microbiota-Immune System)

There is a bidirectional relationship between microbiota and EDCs and since they both have been implicated in metabolic disease pathophysiology, we can assume that this interrelationship is not innocent (54). On the one hand, the GI bacteria with their catalytic enzymatic properties have the ability to metabolize numerous EDCs and, hence, either augmenting or diminishing their toxic effects to the mammalian host, while, on the other hand, EDCs may disrupt the composition and the physiological functions of the GI microbiota, triggering adverse metabolic effects (110). Although we currently do not know the exact underlying mechanisms, it is certain that GI microbiome is a novel regulator of the overall toxicity of EDCs in metabolism.

The role of the immune system in metabolic health has recently drawn the attention of the scientific society. Experimental data are demonstrating that both innate and adaptive immune reactions can crucially affect metabolic disease progression. Since immune cells and cytokine production are physiologically observed in the key organs of metabolism, it is believed that there is sustained co-interaction between the immune system and metabolic tissues (111). Thus, any immune dysfunction can adversely influence metabolic regulation. Simultaneously, experimental data suggest that EDCs can have immunomodulatory properties. For instance, in an experimental model of pregnant female rodents, it was shown that perinatal BPA exposure was accompanied by an imbalance in proinflammatory and anti-inflammatory immune responses, which longitudinally can affect their propensity to disease in adulthood (112). Furthermore, BPA and phthalates can alter cytokine levels, via their estrogen-like properties. Miao et al. showed that rats exposed to BPA displayed reduced expression of ER-a in islets, associated with increased proinflammatory cytokine levels in pancreatic lysates (113). Overall, although data are still indicative, immune dysfunction is highlighted as another unifying mechanism underlying the EDC-associated metabolic disease (114).

### Sex-Dependent Effects of EDCs in the Sexually Dimorphic Metabolism

Human metabolism is characterized by important sex-specific asymmetries. Since the first observation that males and females differ in how they utilize and accumulate fat, a huge progress has been made in the effects of gonadal hormones in metabolic regulation (115). In fact, we currently know that even the central control of energy balance display sex dimorphic traits (116). For example, female POMC system is more responsive to leptin and less responsive to insulin, compared to males, an effect that is mediated through sex steroids (117). Similarly, peripheral metabolic organs, such as liver and adipose tissue, are equipped with estrogen and androgen receptors, which have the ability to alter metabolic signaling pathways in a sexually- dependent way (118, 119).

Apart from the human metabolism itself, EDCs also exert sexually dimorphic effects in metabolism regulation, via direct agonism or antagonism with sex hormone receptors. There is literature evidence that developmental EDC exposure, including BPA, results in altered neurodevelopment as early as fetal life, with sex specific effects observed throughout the brain even before puberty, indicating that the brain, the central regulator of energy homeostasis, is vulnerable to the sex-specific effects of EDCs(120). Furthermore, EDCs have the ability to masculinize or feminize metabolic traits, depending upon their dose and exposure duration. For example, in experimental models, BPA and DES were shown to induce increased body weight in female rodents and decreased or not altered body weight was observed in male ones (121, 122). Finally, in another study it was highlighted that female mice developmentally exposed to BPA exhibited decreased motivation to engage in voluntary physical activity and altered metabolism of carbohydrates, in comparison to males where none of these effects were observed (123).

## Extrapolating Cellular Mechanisms and Effects to Metabolic Disease Pathophysiology

**Figure 3:**
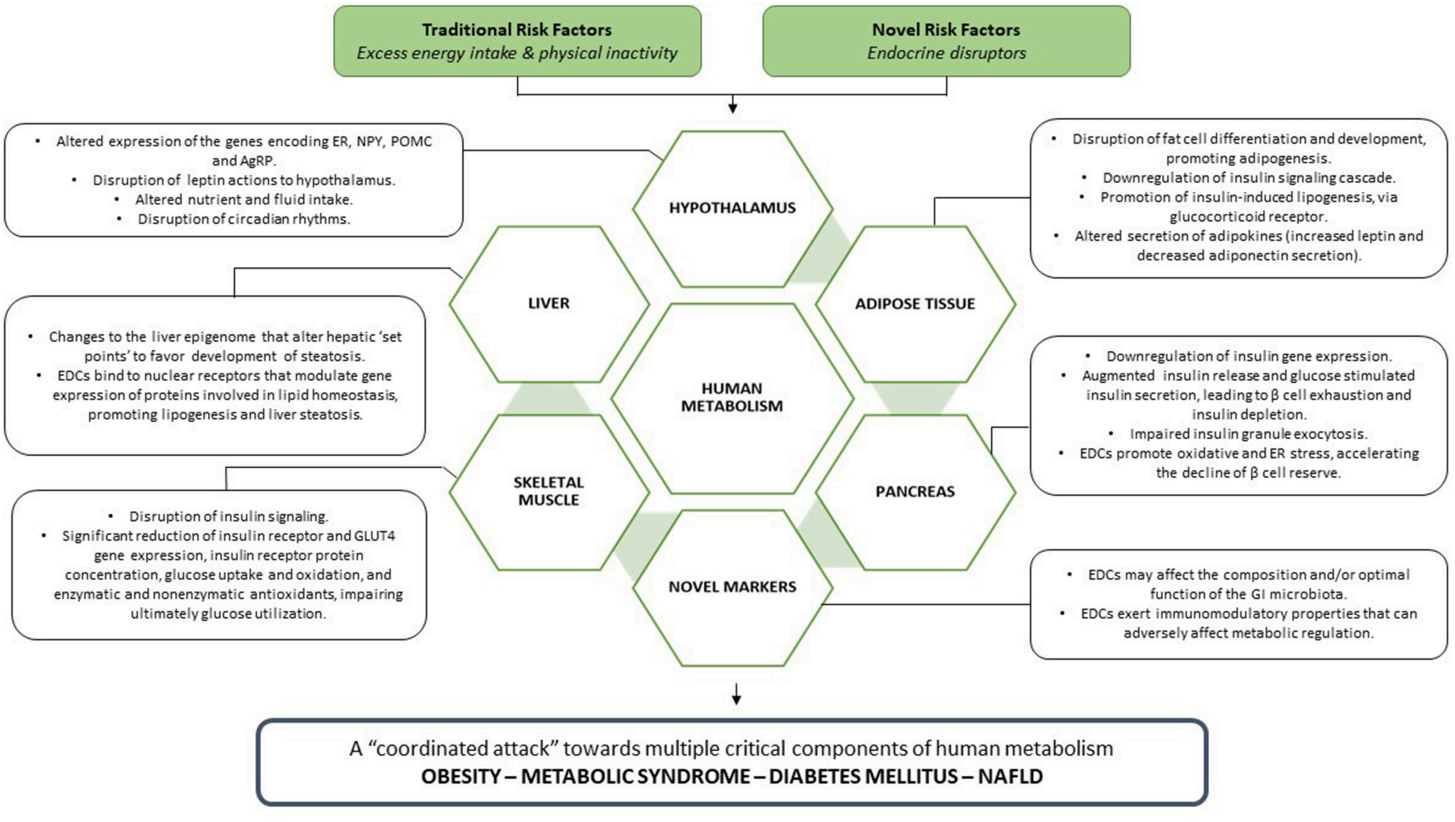
EDCs, acting in parallel with traditional metabolic risk factors, unleash a coordinated attack toward every crucial component of human metabolism, leading ultimately to the manifestation of metabolic disease.

EDCs exert deleterious effects toward multiple critical components of human metabolism (Figure 3), leading to the manifestation of metabolic disease, clinically expressed as obesity, metabolic syndrome, diabetes mellitus, and NAFLD.

- **Obesity:** A subset of EDCs, called “obesogens,” promote adiposity by altering programming of fat cell development, increasing energy storage in fat tissue, and interfering with neuroendocrine control of appetite and satiety. Approximately 20 environmental chemicals are already known to exert obesogenic actions. Although the “obesogen hypothesis” was recently established, the body of evidence we have is enough to better comprehend obesity pathophysiology, in order to proceed to preventive measures. Reducing caloric intake and encouraging physical activity are key factors in tackling obesity. However, limiting EDC exposure, particularly during sensitive, developmental life stages, can be analogously beneficial in limiting the incidence of this burgeoning health problem (124).
- **Insulin Resistance—Metabolic syndrome—Diabetes Mellitus:** Parma Consensus in 2015 has also introduced the “metabolic disruptor” hypothesis, according to which “environmental chemicals can act during development and/or other sensitive time periods across the lifespan to control adipose tissue development and/or by altering food intake and metabolism via specific effects on the brain, pancreas, adipose tissue, liver, GI tract, and muscle individually or in combination”(18). Susceptibility to metabolic disease may originate solely from the EDC exposure. However, in some cases, a second “hit” (e.g., high fat diet, stress) may be necessary for the EDCs effects to be clinically expressed. Either way, current scientific data are indicative that EDCs are implicated in metabolic disease pathogenesis and should be used as a solid ground not only for further research, but also for preventive strategies.
- **Non-alcoholic fatty liver disease (NAFLD)**. NAFLD represents one of the most rapidly rising and most prevalent liver disease worldwide. Its close association with metabolic syndrome and diabetes mellitus has urged scientific community to better understand its pathogenesis (125). EDCs, as mentioned above can promote NAFLD manifestation via directly or indirectly interfering with liver lipogenesis. In addition, developmental EDC exposure can promote epigenetic alterations, inducing metabolic reprogramming of genes that are involved in hepatic lipid homeostasis toward a metabolic set point that promotes NAFLD (104).

## Limitations of EDCs Research—Future Directions for Scientific Community

There is no doubt that scientific community has made a huge progress in EDCs research so far. However, there are still several challenging questions to address, in order to establish solid conclusions regarding the effect of EDCs in metabolic health and disease.

In fact, several systematic reviews and meta-analyses, published during the last decade, have tried to investigate the real magnitude of adverse effects of EDCs in humans. However, the majority of them have failed to reach definite conclusions and establish clear causal links between EDCs and disease (126–128). For example, reviews concerning the effects of BPA in pubertal development and metabolic disease reported conflicting results (129, 130), unable to substantiate any causal link. Inconclusive studies, in humans, are hammering the current position regarding phthalates and obesity (128), triclosan in various health outcomes (127), as well as EDCs and male reproductive disorders (126).

Therefore, the vital question arises: why we cannot still prove the harmful effects of EDCs in humans, despite that there is a plethora of experimental data? Among the answers the methodological one appears to be one of the most complex ones, since human studies of EDCs are methodologically a topic of challenging scientific research, where key limitations are preventing us from properly interpreting the findings and properly designing optimal human studies (131, 132). These key limitations involve low reliability of exposure assessment of EDCs with short half-lives, EDC mixtures, possibility of non-monotonic dose-response relationships, non-existence of an unexposed group, difficulties in measuring exposure during critical periods, and interactions with established risk factors (131).

So how can we override these research limitations, in order to reproduce human models of EDCs exposure? The answer is that we do not know yet. Among the above limitations, mixtures of EDCs are the most complicated issue. During the past decade, studies relating body exposures of multiple EDCs to endocrine disease have been published. For example, concerning metabolism, in an animal model by Ruzzin et al., rat exposure to a mixture of POPs led to the manifestation of insulin resistance, abdominal obesity, and hepatosteatosis, via a robust inhibition of insulin action and promotion of lipogenesis (133). However, further investigating EDC mixtures may contain pitfalls, particularly in the design of a “representative” EDC mixture that would be comparable to environmentally human exposures. Furthermore, taking into account that every human has a unique exposome, it is hardly possible to predict the net effect of EDCs mixture at the individual level in humans (28).

Furthermore, as we mentioned above, EDCs are commonly characterized by non-monotonic dose responses (NMDR), implying that EDCs actions at one dosage do not necessarily predict effects at another. In NMDR curves, increasing EDC dose is not accompanied by increased disease risk, but, on the contrary, disease risk reaches a plateau or even decreases. The underlying mechanisms of these dose-responses can be multiple, including antagonistic effects induced by receptors differing in their affinity, receptor desensitization, negative feedback with increasing doses, or dose-dependent metabolism modulation (23, 134).

Overall, despite the limitations we face in the design of experimental studies of EDCs and the caution required in deducing causality from epidemiological work in humans, most studies do underpin an interrelationship between EDCs exposure and adverse health outcomes.

In this context, we have the duty to continue exploring the effects of EDCs in human body, always with a multidisciplinary approach, as basic, translational, and clinical scientists' co-interaction can be paramount in translating research into clinical knowledge.

Finally, apart from setting research goals, scientific society should also focus on the social impact of EDCs. Familiarizing society with EDCs' harmful properties should be one of the primary focuses of scientific community. Through awakening general public, it might be easier for scientific and international organizations to draw the attention of politicians, who have a legislative role, as well as of regulatory agencies that evaluate EDCs, in order to promote changes in public health policies.

In the future, if we manage to improve research strategies and amplify social vigilance regarding EDCs, we are confident that through the accumulating evidence we will be able to promote greater regulation, more precaution and gradual restriction of the EDCs industrial uses, offering to our offspring a cleaner environment.

## Conclusions

EDCs represent an emerging global threat for human's metabolic health. Scientific data published during the last 10 years have made an exponential progress in better understanding how environmental chemicals spherically attack metabolism, via interfering with every metabolically active organ of our body. Considering the fact that over 1,000 synthesized chemical compounds have been acknowledged as EDCs, it is clear that human metabolism is under a constant and coordinated attack. Although limitations of EDCs research do exist, there is no doubt that it is high time we took action. Improving research strategies, promoting public knowledge, and initiating preventive measures in EDCs industrial uses and applications can be key factors in tackling the global deterioration of metabolic health we currently encounter. The health of future generations is under attack, suggesting that if we don't further explore this scientific area, our children could be affected in decades to come.

## Author Contributions

ED-K designed and supervised the project, devising the main conceptual ideas and proof outline. OP, EK, and GP contributed to the implementation of the project and to the writing of the manuscript. All authors contributed to the final version of the manuscript.

### Conflict of Interest Statement

The authors declare that the research was conducted in the absence of any commercial or financial relationships that could be construed as a potential conflict of interest.
